# Our Experience with Iatrogenic Ureteric Injuries among Women Presenting to University College Hospital, Ibadan: A Call to Action on Trigger Factors

**DOI:** 10.1155/2019/6456141

**Published:** 2019-02-10

**Authors:** Olatunji Lawal, Oluwasomidoyin Bello, Imran Morhason-Bello, Rukiyat Abdus-salam, Oladosu Ojengbede

**Affiliations:** ^1^Genitourinary/Urogynaecology Unit, Department of Obstetrics and Gynaecology, University College Hospital, Ibadan, Nigeria; ^2^Genitourinary/Urogynaecology Unit, Faculty of Clinical Science, UI, Ibadan, Nigeria; ^3^Adeoyo Maternity Teaching Hospital/Fistula Centre Ibadan, Ibadan, Nigeria; ^4^Centre for Population and Reproductive Health, Ibadan, Nigeria

## Abstract

**Background:**

Ureteric injuries leading to ureterovaginal fistula (UVF) is less common than vesicovaginal fistula, as a cause of urinary incontinence. Recently, there is a surge in the number of UVF cases presenting to University College Hospital (UCH) following a caesarean delivery. The urogynaecology unit at UCH is at the forefront of providing surgical repair for women with all forms of genitourinary fistulas. We describe our experience with managing UVF arising from ureteric injury.

**Methods:**

A retrospective data collection of UVF cases managed from January 2012–December 2017 at UCH is presented. Information on sociodemographic and obstetric characteristics, presenting complaints, antecedent surgery, treatment received, findings at surgery, and postoperative complications were obtained with a structured proforma.

**Results:**

Eighteen cases of UVFs due to iatrogenic ureteric injury were managed. Majority (*N*=11; 61.1%) of the women suffered the injury following the emergency caesarean section (EMCS). Abdominal hysterectomy operation accounted for four (22.2%) cases, and one case each (5.6%) was due to vaginal hysterectomy and destructive operations. Prolonged obstructed labour (POL) (81.8%) was the most common indication for the EMCS, while 18.2% had surgery on account of lower uterine segment fibroid. Most of the ureteric injuries were on the left side. Postoperative complications documented were haemorrhage, urinary tract infection, wound infection, and injury to the neighbouring structure.

**Conclusion:**

Caesarean section being one of the most performed surgical operations in Nigeria was surprisingly found to be the most common cause of ureteric injury ahead of hysterectomy. It is a pointer that the surgeons might not have properly learnt the art of the caesarean delivery well. We recommend adequate surgical training of medical officers/surgeons that are involved.

## 1. Introduction

Ureteric injury resulting in ureterovaginal fistula (UVF) is an infrequent complication of abdominopelvic surgery. UVF patients suffer from prolonged hospital stay, delayed postoperative recovery time, and persistent urinary incontinence [1]. This is usually distressing to the patient because of increased morbidity, cost, and repeat surgical interventions when the initial repair fails. The reported prevalence of ureteric injuries appears to be higher in high-income countries than low-middle income countries [2]. The possible explanation is the increasing rate of minimal access surgery in the pelvic region [2].

Minimal access surgery is associated with higher risk of ureteric injury than open surgery [2]. The incidence of ureteric injury varies between 0.5 and 1.5% as a complication of open gynaecologic surgery and 0.5–14% in laparoscopic surgeries in high-income countries with higher prevalence reported among the inexperience surgeons [3]. Surgeons' experience plays a critical role in reported incidence of ureteric injury following laparoscopic gynaecologic surgeries. Some authors reported a higher incidence, while others reported comparable figures between laparoscopic and open surgeries [1, 2]. Majority of these ureteric injuries occurred from laparoscopic-assisted vaginal hysterectomy [3]. The pattern of ureteric injury in low-middle countries was initially reported to be due to gynaecological surgery. However, there is a changing pattern of the trigger factor for UVF with the caesarean section increasingly reported. The incidence in most institutional review in Nigeria varies between 0.3 and 0.45% for both obstetrics and gynaecologic procedures [4, 5]. There are concerns that the caesarean section may contribute more, especially in unreported cases that eventually present in fistula centres [6]. Reported injury to the ureter is highest when a patient has any of the following associated factors: presence of endometriosis, pelvic infection, previous abdominopelvic surgery, and radiation therapy [7]. Other associated risk factors are huge pelvic masses, pelvic malignancy, haemorrhage, and congenital anomalies of the urinary system [7, 8].

The reported clinical presentation of ureteric injury depends on the type of injury to the ureter. Ligation and transection injuries are the most common type of injury usually from gynaecological and obstetrics surgery [9]. Other forms of injury that could occur include thermal injury, kinking, devascularization, partial/complete transection, and perforation. Complete transection of the ureter causes immediate leakage of urine within the first 24–48 hours of surgery, while ligation and thermal injuries present later following tissue necrosis. Intraoperative cystoscopy has improved diagnosis, but in low resource setting, this may not be feasible due to lack of equipment and or skills to perform cystoscopy [10]. However, high index of suspicion with symptoms such as flank pain and fever may suggest ureteric injury after pelvic surgery. There may be frank leakage of urine following ureteric transection. This study was undertaken to describe the emerging pattern of increasing UVF in our centre to showcase avoidable morbidity in obstetric practice.

## 2. Methods

The urogynaecology unit in the Department of Obstetrics and Gynaecology of the University College Hospital was established to improve maternal health by reducing the burden of pelvic floor dysfunction and genital fistula through providing comprehensive quality care for all women. The unit is also providing training and mentorship to the Fistula Care Centre in the department of Obstetrics and Gynaecology, Adeoyo Maternal Hospital, Oyo state.

This retrospective study was conducted at University College Hospital and Adeoyo Maternity hospital after obtaining permission from Oyo State and UI/UCH Ethical committee. Inclusion criteria were patients with ureterovaginal fistula from iatrogenic ureteric injuries. Patients with ureteric fistula from trauma, radiation therapy, and granulomatous infection were excluded.

The unit protocol for managing patients with suspected genital fistula involves a detailed history and examination at first presentation, followed by examination under anaesthesia and dye test in the day case theatre. The genital fistula was properly examined, and clinical details such as site, size, number, affectation of bladder neck, urethra, and degree of vaginal scarring can be assessed without eliciting pain. A negative dye test with leakage of clear urine suggests ureteric fistula. Abdominopelvic scan and intravenous urogram were requested to confirm the diagnosis. Cystoscopic evaluation was performed for patients with suspected combined vesicovaginal and ureteric fistula. The initial evaluation aids in proper planning of the surgical approach and level of expertise needed for repair. The week of surgery; baseline investigations; full blood count, serum electrolyte, urinalysis, urine microscopy, culture, and sensitivity; and clotting profile were obtained. Chest X-ray and electrocardiogram were requested on need basis. Surgery was performed under anaesthesia in the supine position. Type of procedure performed was determined by level of obstruction and type of ureteric injury. A ureteric stent was passed to aid proper healing of the anastomosis and maintained for one week. Routine postoperative care was continued for two weeks after which urethral catheter was removed. Patients were discharged and reviewed in clinic in four weeks and subsequently in three-month interval for six months.

Data were cleaned, entered, and analyzed in statistical package for social sciences version 22, and frequencies and other statistics were calculated and used to summarize tables.

## 3. Results

During the period of the study (January 2012 to December 2017), a total of one hundred and one genitourinary fistula surgeries were performed out of which 18 patients had ureterovaginal fistula from iatrogenic ureteric injury with an incidence of 17.8%. The age of patients ranged between 28 and 80 years (mean 42.7 (SD = 13.5)). Parity of the patients ranged between 1 and 8 children. Majority were multiparous with at least three children.  Table 1 shows the age, parity, religion, level of education, and the marital status of the patients.

Table 2 shows the clinical characteristics of the patients. Most of the patients had urinary incontinence for more than six months, with two patients leaking for 16 years and 18 years, respectively. More than half of the patients had left ureteric injury at surgery, and one patient had injury in both ureters. Four women had combined vesicovaginal fistula and left ureteric fistula.

Majority of the women suffered ureteric injury during emergency caesarean section (*N*=11; 61.1). Abdominal hysterectomy was the second most common cause seen in 4 (22.2%) patients, and vaginal hysterectomy and destructive operation were reported in 1 (5.6%) patient, respectively.

The indication for the emergency caesarean section was prolonged obstructed labour in 9 patients (50.0%) followed by symptomatic fibroid reported in 2 (11.1%) cases. Other indications are shown in Table 2.

Patients were reported to have sustained their injuries at various levels of healthcare. Eight (44.4%) patients sustained their injuries in private hospitals, while the remaining sustained injuries at government-owned hospitals (4 {22.2%} at the tertiary hospital and 1{55.6%} at the state hospital), respectively. All patients had ureteroneocystostomy performed with insertion of a stent. Estimated blood loss ranged from 200.0 to 1200.0 mls. A patient had intraoperative haemorrhage from inadvertent injury to the left internal iliac artery which was successfully repaired by the vascular surgeon. Postoperative 2 patients had grade 2 wound infection which was treated with antibiotics with daily wound dressing, and 1 patient had postoperative pyrexia from upper respiratory tract infection, while another had urinary tract infection which resolved with antibiotics therapy. We recorded 100 percent success of surgical repair and at discharge from the hospital.

## 4. Discussion

This study reviewed the clinical factors associated with ureteric injury in patients managed in a newly designated fistula care centre in Nigeria. Emergency caesarean section from prolonged obstructed labour accounted for the majority of the injury, and the left ureter was the most common affected side. Majority of the women had their caesarean section at the private hospital, and these surgeries were performed by the medical personnel and other cadres of health workers that are not licensed to perform surgery.

In Nigeria, vesicovaginal fistula is the most prevalent form of genital fistula from prolonged obstructed labour and entrapment of the fetal head between the maternal pelvic bones and soft tissue leading to ischaemia and pressure necrosis of the bladder and vagina [11]. In this study, the most common cause of ureteric injury presenting to our fistula centre was due to surgical mishap during caesarean sections. This finding is similar to a previous review in a tertiary health institution in Eastern Nigeria in which the obstetric procedure was documented as the leading cause of ureteric injuries [5].

Although we could not retrieve any publication on ureteric fistulas from other fistula centres in Nigeria, a recent review by Engender Health reported increasing incidence of iatrogenic ureteric injuries following the caesarean section prompting a call to action [12]. The injury usually occurs during difficulty in securing haemostasis from a lateral extension of the lower transverse incision of the uterus during delivery of an impacted fetal head or during caesarean hysterectomy to arrest intractable postpartum haemorrhage [6, 13]. Other reports from Nigeria focused on ureteric injuries from gynaecologic surgery. For example, Onwudiegwu et al. reported that ureteric injuries occur more commonly from hysterectomy like previous reviews from other parts of the country [4, 9, 14, 15].

In the patients under study, the left ureteric injury occurred frequently following surgery. The same trend was seen in studies conducted in other institutions [5, 6, 14]. The predilection for the left ureter is not fully understood, but explanation given includes the variable path taken by the left ureter bringing it closer to the pelvic structures, positioning of a right-handed surgeon during surgery when the surgeon predisposes the left ureter more to iatrogenic injures [6]. While these theories cannot be proven, no ureteric injury has been associated with either obstetrics or gynaecology procedure based solely on the side affected. In our own cases, all left ureteric injury occurred from the emergency caesarean section. More reviews may need to be done in other centres to confirm if a pelvic procedure increases injury risk to left compared to the right ureter.

Injury to the ureter takes many forms, from ligation, transection, devascularization, crush, and perforation. Ligation was the most common injury in our review. Similar review conducted in eastern Nigeria found ligation and transection the most common form of injury [9, 16]. This could also be explained by the surgeons attempt to secure haemorrhage during surgery, and poor reflection of the bladder flap from the uterus during repair of the uterine angle during the caesarean section. Ligation could also account for the delayed presentation in most patients. Leakage of urine and ureterovaginal fistula develops late, 10–14 days after the procedure compared to transection which may be immediate or earlier in the postoperative period.

**Figure 1:**
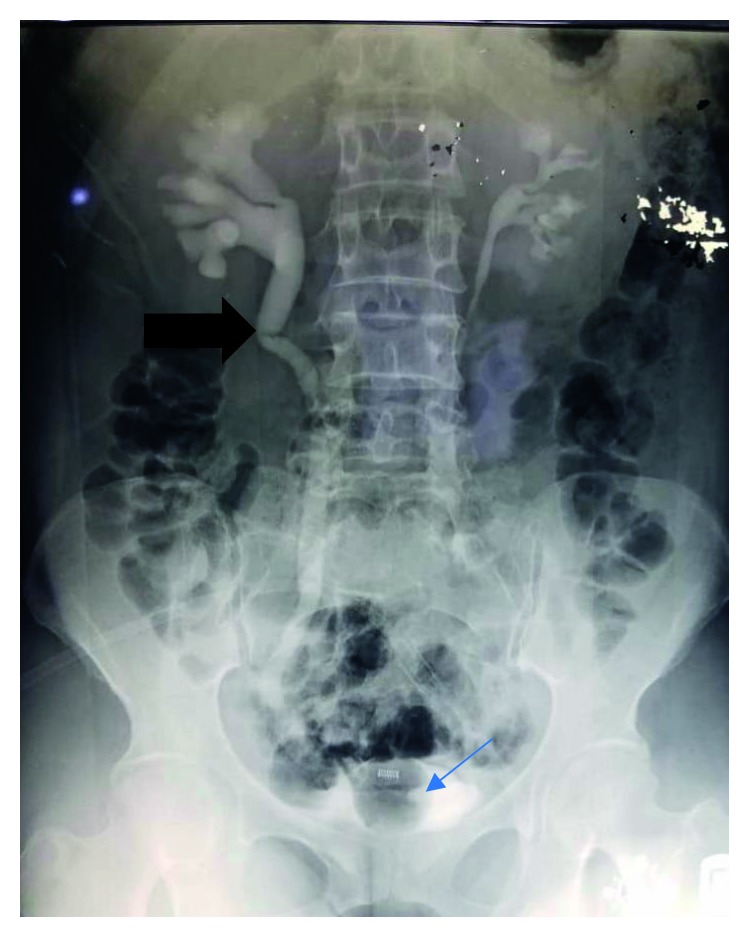
Intravenous urogram showing grade II right hydronephrosis with hydroureter (large arrow) and left ureterocele (small arrow).

Diagnosing ureterovaginal fistula from ureteric injury requires a combination of history, examination, and imaging. Leakage of urine through the vagina was the main complaint of our patients in this study. While most of the patient retained the ability to micturate despite incontinence a few patients had total incontinence due to a combined vesicovaginal fistula. This makes the diagnosis more confusing, and closure of the vesicovaginal fistula alone will not stop the incontinence. Intravenous urography and retrograde pyelography are the imaging of choice for diagnosis of ureteric fistulas. However, they are expensive and not readily available in some low resource centre. A study in northern Nigeria diagnosed ureteric injury by three three swab test and found it to be useful in low resource settings as an alternative to advance imaging [17]. The drawback of a dye test is the inability to identify which ureter is involved. In our centre, most of our patients had an IVU to confirm diagnosis. Findings from imaging studies connoting ureteric injury includes presence of hydronephrosis (Figure 1) and periureter flare from leakage of urine around the ureter [18]. Also, imaging helps to the assess the level of obstruction and status of the contralateral ureter and bladder.

The few patients who had a combined fistula were discovered intraoperatively, following successful closure of the vesicovaginal fistula and persistent leakage of clear urine from the vagina. The intraoperative dye test done showed no leakage of dye from the vagina, a likely ureteric fistula was suspected, and repair was initiated at the same surgery. A coexisting vesicovaginal fistula and ureteric fistula in our environment reflects the progression of injury in patients who had suffered both a prolong obstructed labour and pressure necrosis and trauma from surgical intervention causing ureteric fistula.

The aim of genital-fistula repair is to restore continent and other reproductive functions. In our unit, before undertaking repairs, urosepsis is excluded with a urine microscopy and culture, and patient optimization is done. In cases with nutrition deficiency and limb deformity, the dieticians and physiotherapist are invited to commence rehabilitation before and after surgery. Immediate or early repair is advocated by most authors with good postoperative outcome [19], especially for cases identified intraoperatively. In cases where early repair cannot be performed either due to sepsis, pelvic abscess, or extensive haematoma formation, relieving obstruction through a ureteric stent for partial transection or ligation and percutaneous nephrostomy is advocated [19].

Complete resolution of symptoms occurs without open surgery in some cases with these procedures. A study in India reviewed interventions for ureterovaginal fistula in 17 patients and concluded that iatrogenic ureterovaginal fistula can be managed successfully by ureteroscopic stenting [20]. Similar finding was also reported in another study where 29 out of 30 patients were successfully managed using the same procedure [21]. All our patients presented late, following the consequence of ureteric injuries with genital fistulas. Intraoperative findings were extensive fibrosis around the area where the affected ureter was ligated, and in a case, the fibrosis extended to the left internal iliac artery. The affected ureter was dissected, and ureteroneocystostomy was performed in all the patients except one who had a Boari's flap.

Different modes of treatment and approach have been tried and found to be successful. Laparoscopic ureteroneocystostomy has an advantage of reducing blood loss, early recovery, less postoperative pain, and successful outcome [22]. Boari's flap and psoas hitch are recommended to reduce tension at the anastomosis for ureters in which a long segment have been lost due to devascularization or transection [23]. Some authors have successfully used the appendix or ileum as the interposition graft and transureteroureterostomy [24]. Some studies have reported a high success rate with ureteroneocystostomy alone compared to other methods [20]. Complication associated with ureteroneocystostomy includes failed anastomosis leading to extravasation of urine or persistent fistula formation and ureter stricture at point of anastomosis [21]. All the patients had a successful repair, and one patient had injury to the left internal iliac vein which was successfully repaired by the cardiothoracic surgeon. Other complications seen in our patients were wound infection, upper respiratory tract infection, and urinary tract infection which responded to antibiotics.

Preventing ureteric injury during pelvic surgery is key in reducing the morbidity associated with ureterovaginal fistula. Preventive measures start from adequate patient preparation. Preoperative imaging to delineate the course of the ureters, good anatomic knowledge of the course of the ureter, proper dissection and avoidance of indiscriminate clamping, and use of electrocautery on the blood vessel may help reduce injury to the ureter. In our study, most injuries occurred from obstetric complications. We recommend the use of partograph and timely intervention with caesarean section performed by skilled surgeons to prevent complications from prolonged obstructed labour. The need to train care givers on alternative methods of providing vaginal delivery for dead fetuses rather than caesarean delivery may also be prudent.

Limitation to the study includes our inability to perform renal scintigraphy especially in those that had ureteric injuries for many years before presentation. Also, the cadre of health professionals who performed the antecedent surgery could not be ascertained since most were performed in private hospitals. There could also recall bias on the events that led to the injury.

## 5. Conclusion

Ureteric injuries presenting as ureterovaginal fistula may be uncommon, but it is becoming more distressing on seeing women suffering from these injuries due to the caesarean section. This mishap call for action to review surgical training of medical officers/surgeons that are involved. We recommend emergency obstetric care training with hands-on experience in the emergency caesarean section. Above all, early diagnosis of poor progress of labour might also avert the danger of delivering impacted head of the fetus.

## Figures and Tables

**Table 1:** Sociodemographic characteristics.

Variable	Mean (SD)	
Age	42.7(13.5)	
Variables	Frequency (*n*)	Percentage
*Parity*		
0–1	01	5.60
2–4	14	77.9
>5	03	17.8
*Religion*		
Islam	11	61.1
Christianity	07	38.9
*Level of education*		
Primary	15	83.3
Secondary	02	11.1
Tertiary	01	5.60
*Marital status*		
Single	None	0
Married	03	16.6
Separated	10	55.6
Divorced	05	27.8
*Occupation*		
Skilled	4	22.2
Unskilled	14	77.8

**Table 2:** Clinical features.

Variables	Frequency (*n*)	Percentage
*Duration of leakage*		
>6 months	06	33.3
6 months–<1 year	06	33.3
>1 year	06	33.3
*Affected ureter*		
Left ureter	12	66.7
Right ureter	05	27.8
Both	01	5.6
*Antecedent surgery before injury*		
Emergency caesarean section	12	61.1
Abdominal hysterectomy	04	22.2
Vaginal hysterectomy	01	5.6
Destructive operation	01	5.6
*Indication for antecedent surgery*		
Prolonged obstructed labour	11	50.0
Symptomatic uterine fibroid	02	11.1
Uterine prolapse	01	5.6
Endometrial cancer	01	5.6
Abortion	01	5.6
CIN	01	5.6
Failed VBAC	01	5.6
*Hospital setting of antecedent surgery*		
Private	12	44.4
General	02	5.6
Tertiary	04	22.2
*Anaesthesia*		
General anaesthesia	03	16.7
Subarachnoid block	11	61.1
Epidural block	04	22.2
*Type of repair*		
Ureteroneocystostomy	17	94.4
Boari's flap	01	0.56
*Complications (n*=8)		
Wound infection	06	66.8
URTI	01	11.1
UTI	01	11.1

## Data Availability

The data used to support the findings of this study are available from the corresponding author upon request.
